# Determinants of Adherence to ADA Type II Diabetes Mellitus Guidelines: Implications for Longevity

**DOI:** 10.1093/geroni/igaa057.725

**Published:** 2020-12-16

**Authors:** Arseniy Yashkin, Igor Akushevich, Anatoliy Yashin

**Affiliations:** Duke University, Durham, North Carolina, United States

## Abstract

The aim of this study was to identify the differences in terms of demographics, socioeconomic status and overall levels of morbidity-related health burden between population strata characterized by high levels of adherence to American Diabetes Association screening guidelines and their low-adherence counterparts. Factor analysis was used to create a single continuous measure of adherence which was stratified and analyzed using the Cox proportional hazards model to identify adherence levels associated with protective effects for mortality. Based on the results, the entire population of Health and Retirement Study respondents newly diagnosed with diabetes mellitus, type II was then stratified into four levels of adherence – excellent, sufficient, insufficient, poor – based on the strength of the protective effect associated with that level of the adherence factor and compared. Mortality in the group associated with excellent adherence was 41 to 57 percentage points lower than among their counterparts. High levels of adherence were associated with White and Hispanic race, low morbidity burden, high education and economic status, and low levels of functioning limitations. Based on race-specific survival function estimates we found that the life expectancy at age 65 of an individual newly diagnosed with type II diabetes mellitus could be improved from 14.97 to 19.64 years for whites, 13.36 to 19.58 years for African Americans and 14.92 to 21.28 for Hispanics if average adherence levels are increased to the highest levels observed in our study. Finally, we found that adherence levels were improving over the 1991-2015 period suggesting successful diabetes awareness efforts.

